# Neutrophil-to-Lymphocyte Ratio (NLR) in Canine Inflammatory Bowel Disease (IBD)

**DOI:** 10.3390/vetsci7030141

**Published:** 2020-09-22

**Authors:** Elena Benvenuti, Alessio Pierini, Eleonora Gori, Claudia Lucarelli, George Lubas, Veronica Marchetti

**Affiliations:** Department of Veterinary Sciences, University of Pisa, Via Livornese Lato Monte, 56121 Pisa, Italy; elebenve81@gmail.com (E.B.); pierini.alessio2004@libero.it (A.P.); lucarelli.claudia@gmail.com (C.L.); george.lubas@unipi.it (G.L.); veronica.marchetti@unipi.it (V.M.)

**Keywords:** dog, IBD, inflammation, leukocytes, clinical response

## Abstract

Inflammatory bowel disease (IBD) is a multifactorial chronic inflammatory disorder leading to structural changes in the intestinal wall. In humans, the neutrophil-to-lymphocyte ratio (NLR) has been proposed as a promising marker of IBD. This study evaluated the possible clinical and prognostic significance of the NLR in dogs with IBD. This retrospective study enrolled 41 dogs diagnosed with IBD presented to University of Pisa from January 2017 to January 2018. For each dog, age, sex, canine chronic enteropathy clinical activity index (CCECAI), endoscopic and histopathological grading were recorded. Complete blood count, serum total protein, albumin, cholesterol, and C-reactive protein at the time of endoscopy were recorded. A control group (CG) of healthy dogs from a blood donor database was built. NLR was calculated for both IBD and CG as the ratio between absolute neutrophils and lymphocytes. Presence of crypt distension, lacteal dilation (LD), mucosal fibrosis, intraepithelial lymphocytes was recorded. Follow-up information was obtained from electronic medical records and dogs were classified as responders and non-responders based on CCECAI variation between admission and the first recheck. IRE dogs showed higher NLR compared to healthy dogs. NLR correlated negatively with total protein, albumin, and cholesterol and correlated positively with CCECAI. Dogs with LD showed higher NLR than dogs without LD. Non-responders showed higher NLR compared to responders. In conclusion, as in IBD human patients, the NLR acts as an inflammatory marker providing further information on severity of the disease and could be useful in predicting treatment response.

## 1. Introduction

Chronic enteropathies (CE) can be subdivided retrospectively in relation to the response to treatment into food-responsive enteropathy (FRE), antibiotic-responsive enteropathy (ARE), immunosuppressant-responsive enteropathy (IRE) [1].

The definition of canine inflammatory bowel disease (IBD) identifies an intestinal idiopathic inflammation that implies failed treatment trials with diet and antibiotics. In IBD, intestinal inflammation has to be demonstrated by histopathological examination and an immunosuppressant/steroid therapy is needed [1,2,3]. The term IBD is also used in veterinary medicine interchangeably with IRE in cases not responding to diet or antibiotic [4].

Several studies have attempted to identify the prognostic factors of IBD. These include two clinical scoring systems [5,6], hypocobalaminemia [7], hypoalbuminemia [8], serum C-reactive protein (CRP) [5], and a high canine pancreatic lipase immunoreactivity concentration [9]. Since the classification of canine CE is still changing and therapy has still not been standardized, clinicians are searching for other good predictive markers to classify IBD-affected dogs in different risk classes.

The neutrophil-to-lymphocyte ratio (NLR) has been used in human medicine as an easily accessible parameter that can be calculated using a white blood count differential [10]. The NLR has been identified as a predictor of mortality in oncological and cardiopathic human patients [10,11,12]. In humans, the NLR has also been studied in patients with IBD, in which the NLR seems to be higher in patients with active diseases compared to inactive forms [12,13,14,15,16,17].

To the best of our knowledge, there are no specific studies on the NLR in canine IBD, as it has been evaluated only in oncological patients and in patients affected by septic peritonitis and systemic inflammatory response syndrome [18,19,20,21,22,23]. The aim of the study was therefore to evaluate the possible clinical significance of the NLR in dogs with IBD.

## 2. Materials and Methods

This retrospective study was performed by searching the medical databases of University of Pisa in order to find dogs with a diagnosis of IBD between January 2017 and January 2018. Since this study involved a retrospective medical records review, formal ethical approval was not necessary. However, a written informed consent is always obtained from each owner for the use of clinical data for scientific purposes.

IBD was diagnosed after the exclusion of extraintestinal diseases, endocrinopathies, infectious or parasitic diseases, and intestinal disease of other etiology (e.g., mechanical obstruction from intussusception, foreign body, or intestinal tumors). All dogs had full hematobiochemical panel including bile acids, serum trypsin-like immunoreactivity, canine pancreatic lipase, serum cobalamin, basal cortisol or adrenocorticotropic hormone (ACTH) stimulation test, and urinalysis with urinary protein-to-creatinine ratio. In addition, all dogs had a full abdominal ultrasound performed by three radiologists belonging to the referral center (University of Pisa) at the time of the inclusion in the study. Furthermore, dogs were included in the IBD group after the exclusion of food responsive enteropathy (FRE), responding to a hydrolyzed diet for at least of 2 weeks. Although included dogs that were referred to the study centers had all already done dietary trials with highly digestible or monoprotein diets without clinical benefit, dogs were again subjected to a diet trial with a hydrolyzed diet. In all dogs, during diet trial a multistrain probiotic was used. The antibiotic responsive enteropathy (ARE) was excluded through antibiotic trial with Tylosin at 10 mg/kg q12h for 3 weeks. Dogs with FRE and ARE were excluded from the study.

Electronic medical records were searched for a control group of healthy dogs (CG group). Each dog included in the control group was a member of the blood donor program of the University of Pisa. Blood donor dogs were selected using the criteria indicated in the Ministry of Health Italian Guidelines [24]: age range from 2 to 8 years, weight > 25 kg, regularly vaccinated, and that have not traveled outside Italy or received a blood transfusion previously. At the time of donation, each animal is classified as healthy after an evaluation of history, physical examination, hematobiochemical profile, urinalysis (including protein to creatinine ratio), and coprological exam. In addition, each dog had to have negative serological ELISA test for *Leishmania infantum*, and ImmunoFluorescence Antibody test for *Anaplasma phagocytophylum*, *Babesia* spp., and *Ehrlichia canis*.

For each dog, data regarding age, sex, canine chronic enteropathy clinical activity index (CCECAI) [5], WSAVA endoscopic and histopathological grading [2] were collected. The NLR was calculated for both healthy and IBD dogs as absolute segmented neutrophils plus band neutrophils divided by absolute lymphocytes. In enteropathic dogs, NLR was evaluated at the time of the endoscopy and before immunomodulatory treatment (T0).

In addition, complete blood count (CBC), serum total protein, serum albumin, cholesterol, and C-reactive protein (CRP) at the time of the endoscopic procedure were recorded. Dogs with a serum albumin < 2.7 g/dL were diagnosed with protein-losing enteropathy (PLE) [25].

In addition, following Jergens et al. [26], the presence of crypt distension (CD), lacteal dilation (LD), mucosal fibrosis (MF), and intraepithelial lymphocytes (IELs) in histopathological exam were also recorded [3,26].

The immunomodulatory therapy was based on prednisolone (from 0.5 to 1 mg/kg q12h) or budesonide (3 mg/m^2^ q24h), and in some dogs they were associated with cyclosporine to 5 mg/kg q24h or chlorambucil 2–4 mg/m^2^ q24h at the discretion of the clinician. No patients underwent immunosuppressant therapy during the 3 weeks prior to endoscopy.

Follow-up information was obtained from the electronic medical records database. For the evaluation of the follow-up at 1 month (T1), dogs were divided into “responders” and “non-responders” based on the CCECAI scores at T1. Dogs with a reduction of CCECAI and CIBDAI greater than 25% were classified as responders, whereas dogs with a reduction of CCECAI and CIBDAI < 25% or dogs who died, were classified as non-responders [27].

For each continuous variable the D’Agostino-Pearson test was applied to assess distribution (age, NLR, total protein, albumin, cholesterol, CRP, CCECAI). Normally distributed data were shown as mean ± standard deviation, whereas non-normality distributed data were shown as median and range. NLR was evaluated between CG and IBD dogs using Mann-Whitney U-test. The NLR was correlated with all the parameters reported above (total protein, albumin, cholesterol, CRP, CCECAI) using Spearman’s correlation test (*rho*; *r*). The NLR was also compared between histological (score 1, 2, and 3) and endoscopic scores groups (score 2 and 3) using the Kruskal-Wallis test and Mann-Whitney U-test, respectively. The NLR was also evaluated in relation to the presence or absence of CD, MF, IELs, and LD using the Mann-Whitney U-test. A Receiver Operating Characteristics (ROC) curve was then built only for significant differences in the NLR between groups. Finally, differences in the NLR between responders and non-responders were assessed using the Mann-Whitney U-test. Data were analyzed with Graphpad Prism 7 for Windows (La Jolla, CA, USA) and a *p*-value < 0.05 was considered significant.

## 3. Results

Forty-one dogs with IBD were retrospectively included in the study. Twenty-seven dogs (65.8%) were male (two neutered), and 14 dogs (34.2%) were female (six spayed). Several breeds were included: German Shepherd (*n* = 7), Dachshund (*n* = 2), Boxer (*n* = 2), Rottweiler (*n* = 2), Jack Russel Terrier (*n* = 2), Basenji (*n* = 1), Bolognese (*n* = 1), French Bulldog (*n* = 1), Pug (*n* = 1), Cavalier King Charles Spaniel (*n* = 1), Cocker Spaniel (*n* = 1), Dobermann Pinscher (*n* = 1), Labrador Retriever (*n* = 1), Maltese (*n* = 1), Switzerland Shepherd (*n* = 1), Whippet (*n* = 1), Russian Toy (*n* = 1), English Setter (*n* = 1), and Weimaraner (*n* = 1). Twelve dogs were mixed-breed. The median age was 4 years (range 1–15 years). The descriptive statistics of all the continuous variables (NLR, total protein, albumin, cholesterol, CRP) taken into account in the present study are reported in Table 1. Twenty out of 41 dogs (48.7%) had PLE. Median CCECAI at T0 was 6 (range 2–13). Fifteen dogs (36.5%) had endoscopic score 1, while the remaining 36 dogs had endoscopic score 2. Four dogs had histological score 1, 24 dogs had histological score 2, and 13 dogs had histological score 3.

The control group was composed by 150 healthy dogs admitted in the same study period of IBD dogs. Age and sex were not different between the study groups.

**Figure 1:**
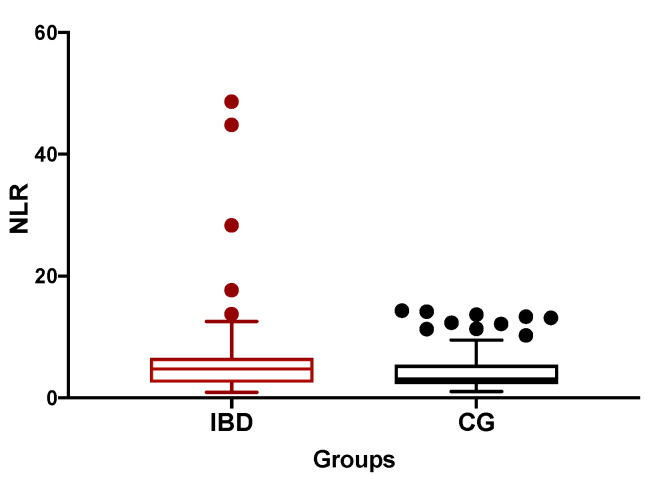
The median neutrophil-to-lymphocyte ratio (NLR) of inflammatory bowel disease (IBD) dogs was significantly higher than control dogs (CG) (4.78, range 0.93–48.64 vs. 3, range 1.1–13.3; *p* = 0.04).

**Figure 2:**
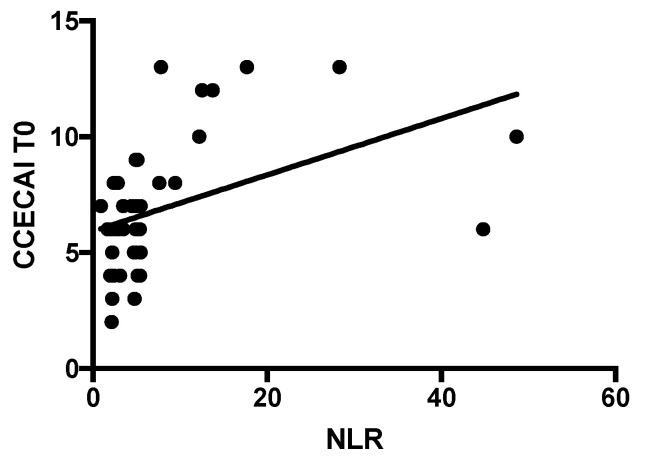
Correlation between NLR and canine chronic enteropathy clinical activity index (CCECAI) score at the admission (*p* = 0.0004; *r* = 0.528).

**Figure 3:**
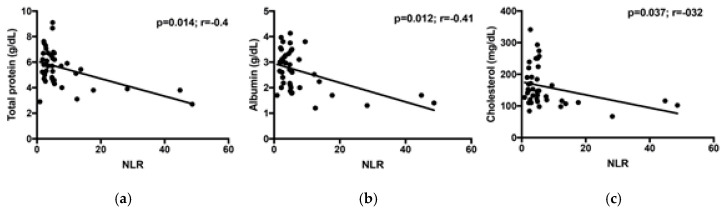
Correlation between NLR and total protein (**a**), albumin (**b**), and cholesterol (**c**) of IBD dogs.

The median NLR of IBD dogs was significantly higher than CG dogs (4.78, range 0.93–48.64 vs. 3, range 1.1–13.3; *p* = 0.04; Figure 1). The NLR correlated moderately and positively with the CCECAI score at T0 (*p* = 0.0004; *r* = 0.528; Figure 2). In the majority of our dogs (31/41), neutrophils are in the reference interval (3700–12,000 K/μL), with one dog with neutropenia and the remaining nine dogs with neutrophilia. Furthermore, 39/41 dogs had lymphocyte count in the reference interval (700–5100 K/μL), while only two dogs (the “outliers” in Figure 2) showed lymphopenia. The NLR correlated negatively with total protein, albumin, and cholesterol (*p* = 0.014, *r* = −0.4; *p* = 0.012, *r* = −0.41, and *p* = 0.037, *r* = −0.32, respectively; Figure 3). The NLR and CRP showed no significant correlation (*p* = 0.9). The NLR was not significantly different either between endoscopic score groups, or between histological score groups (*p* = 0.54 and *p* = 0.38, respectively).

Among the four morphologic features, CD was present in 28 dogs (68.3%), IELs were increased in 16 dogs (39%), and MF and LD were present in 17 (41%) and 22 dogs (53.6%), respectively. However, on the basis of the individual morphological aspects of the histopathology, dogs with LD had a significantly higher NLR than dogs without LD (5.15 vs. 3.29; *p* = 0.04). The ROC curve of the NLR showed that a value of 3.96 was the best cut-off for LD detection with a sensitivity of 82.3% and specificity of 58.3% (*p* = 0.04). The NLR was not statistically different in dogs with or without CD, MF, and IELs (*p* = 0.1, *p* = 0.95, and *p* = 0.7, respectively).

After 1 month after diagnosis (T1), five (12.1%) dogs were considered non-responders and 36 dogs (87.8%) were considered responders. The median NLR was significantly different between responders and non-responders (4.58 vs. 12.23; *p* = 0.009).

## 4. Discussion

In human medicine, many studies have taken the NLR in IBD patients into account, especially in patients with ulcerative colitis [13,14,15,16,17]. In all these studies, the NLR was higher in patients with active UC compared with healthy people and inactive ulcerative patients [13,14,15,16,17]. However, none of these studies found a correlation or association with disease severity.

To the best of our knowledge, this is the first study on the evaluation of the NLR in dogs with IBD. Neutrophils and lymphocytes play different roles in inflammation [28] and may have specific modifications during IBD. During inflammatory states the neutrophil count may increase, while the lymphocytes count may decrease due to inflammation or “stress” leukogram [28]. In addition, we hypothesized that lymphocyte count may decrease due to possible LD in IBD dogs. Consequently, the evaluation of NLR may merge information provided by these two leukocytes and could add some information in this type of patients. Our study showed that IBD dogs had higher NLR values (median 4.78) compared to our healthy dog group (median 3) and compared to the previously published range in healthy dogs (median 2.7) [29]. However, due to a gap in the current literature on the topic, the potential impact of stress itself on NLR cannot be established and these changes may be caused by both disease-linked stress or degree of intestinal inflammation [8,17]. However, in both of these situations, which may be simultaneously present in a dog with IBD, the prognostic role of NLR may be useful.

Clinical monitoring of chronic enteropathies in dogs is currently performed using the CCECAI score [6]. Interestingly, in our study there was a moderate positive correlation between the NLR and CCECAI at T0. This result emphasizes our hypothesis on the possible use of the NLR as a marker of the disease severity in canine IBD. In human IBD, neutrophils are one of the most important infiltrating leukocytes, and are possibly major contributors in the development of tissue injury and inflammation in IBD [13]. As highlighted by Zahorec [10], lymphocytopenia and neutrophilia are a part of the physiological response of the immune system to systemic inflammation and stress. Neutrophils play a key role in the active inflammatory response to stress and they are closely connected to destructive tissue cascades by the secretion of cytokines. On the other hand, previous studies in patients with IBD have highlighted that their lymphocyte function is abnormal both in the circulation and at the mucosal level [13].

Based on our results, the NLR appears to be negatively correlated with total protein, albumin, and cholesterol and to be higher in PLE dogs. Generally, the inflammation of the intestinal tract is more severe during PLE, in which it is common to have a decrease in the above-mentioned biochemical parameters [30]. The PLE can be determined above all by inflammation (lympho-plasmocytic, eosinophilic, granulomatous, and sometimes neutrophilic) and/or lymphangiectasia, which is a lymphatic disorder often secondary to the inflammation itself [30]. According to Equilino and colleagues [30], dogs with PLE have a more severe intestinal inflammation and more pronounced hypoalbuminemia and hypoproteinemia compared to dogs with FRE. Severe forms of intestinal inflammation can be associated with bacterial translocation, local necrosis, and strong systemic complications that may be associated with an increase in neutrophils [31]. In addition, in PLE, a high NLR can also be determined by the loss of CD4+ T cells through retrograde drainage of the lymphatic system in the intestinal lumen [32] and the lymphopenia in lymphangiectasia is thought to be due to loss of lymphocytes through rupture of lacteals [4]. In our study, there was a significant association between the NLR and LD irrespectively of the presence of PLE because 14 dogs had histological LD without hypoproteinemia. Our results thus indicate that the NLR increases in association with intestinal protein loss regardless of the presence of histological lymphangiectasia. A possible explanation could be the systemic neutrophilic response as a reaction to bacterial translocation, caused by the loss of the intestinal mucosal barrier [33]. In IBD dogs, reduced enzyme expression, atrophy of the villi, diffuse infiltration in the lamina propria and in the epithelium with an increase in the number of inflammatory cells and hyperplasia of goblet cells have also been observed [33]. At the moment, it is not possible to exclude that the same mechanisms that happen in humans are present also in dogs.

Finally, we analyzed the NLR in relation to the response to treatment after 1 month, and it seems that the NLR could be useful in predicting the clinical response. It is not fully clear how the severity of inflammation and the type of inflammatory intestinal infiltrate may influence the clinical response.

In our study population, the NLR was not associated with the intestinal or systemic inflammation and the only histological parameter associated with NLR was LD. We cannot exclude that clinical response may be mainly linked to immunological response ability of each singular dog. The latter can then lead to the infiltration of bacteria and their components in the intestinal wall and to the recruitment of neutrophils, thus increasing inflammation. In Celikbilek et al. [14], an increased NLR in active UC patients confirms the key role of neutrophils in the inflammation process [14]. In addition, since PLE can have a worse outcome than no-PLE [31], and in our study PLE patients had a higher NLR, it would be reasonable to think that the NLR may be related to the potential lack of response in PLE dogs.

This study presents some limitations. Although, the number of included dogs was not very low (*n* = 41), larger studies could give more statistical power to our findings and maybe other associations would be found. In addition, it would be interesting to monitor the NLR at the 1-month follow-up in relation to all the other clinical and clinicopathological parameters. Furthermore, although the therapeutic treatment of our study population was very similar (e.g., diet, probiotics, prebiotics, Tylosin), we were unable to standardize the IBD therapy in terms of duration, dosage, and drugs used (prednisolone, cyclosporine, and/or chlorambucil). Another limitation is linked to the small sample size, especially the low number of non-responders, which has to be increased in further studies. In our retrospective study, the presence of ARE was ruled out using an antibiotic trial. Recently, the usefulness of antibiotic trials after diet trials was called into question by Cerquetella et al. [34], who proposed novel inclusion criteria in the diagnosis of IBD, and thus we cannot exclude that the antibiotic trial may have influenced our results. In addition, we cannot predict how other subclinical causes of inflammation (e.g., periodontitis, dermatitis) or therapies may have influenced the NLR. Another limitation is linked to the retrospective nature of the present work, which forced us to exclude dogs with incomplete medical records.

## 5. Conclusions

In veterinary medicine, we believe that this is the first study on the NLR in dogs with IBD. This study demonstrates how an increase in the NLR was associated with more severe forms of enteropathy, with intestinal protein-loss and with the presence of duodenal lymphangiectasia. Although further studies on NLR are warranted, especially in other patients with chronic diseases, as in human medicine, the NLR can be considered both as a useful inflammatory marker and a marker of clinical response for dogs with IBD.

## Figures and Tables

**Table 1:** Descriptive statistics of the continuous variables included in the study (albumin, C-reactive protein (CRP), cholesterol, and NLR).

	Albumin ^1^	CRP ^2^	Cholesterol ^2^	NLR ^2^
Minimum	-	0	67	0.93
25% percentile	-	0.1	115	2.53
Median	-	0.4	145	4.78
75% percentile	-	0.75	190.5	6.56
Maximum	-	2.7	341	48.64
Mean	2.66	-	-	-
Std. deviation	0.79	-	-	-

^1^ Normal distribution after D’Agostino–Pearson normality test and are presented as mean and standard deviation; ^2^ non-normal distribution after D’Agostino–Pearson normality test and are presented as median, minimum-maximum, and 25th–75th percentile.
